# Updating the global occurrence of *Culicoides imicola*, a vector for emerging viral diseases

**DOI:** 10.1038/s41597-019-0197-0

**Published:** 2019-09-30

**Authors:** Samson Leta, Eyerusalem Fetene, Tesfaye Mulatu, Kebede Amenu, Megarsa Bedasa Jaleta, Tariku Jibat Beyene, Haileleul Negussie, Darren Kriticos, Crawford W. Revie

**Affiliations:** 1 Addis Ababa University, College of Veterinary Medicine and Agriculture, P. O. Box 34, Bishoftu, Ethiopia; 2 National Animal Health Diagnostic and Investigation Centre (NAHDIC), P. O. Box 04, Sebeta, Ethiopia; 3 Center for Outcome Research and Epidemiology, Kansas State University, Manhattan, Kansas USA; 4 Commonwealth Scientific and Industrial Research Organisation (CSIRO), GPO Box 1700, Canberra, ACT 2601 Australia; 5 InSTePP, University of Minnesota, St. Paul, MN USA; 6 Department of Computing and Information Sciences, University of Strathclyde, Livingstone Tower (14.01), 26 Richmond Street, Glasgow, G1 1XQ Scotland UK

**Keywords:** Entomology, Viral infection, Ecological epidemiology

## Abstract

*Culicoides imicola* is the main vector transmitting viruses causing animal diseases such as Bluetongue, African Horse Sickness, and Schmallenberg. It has become widely distributed, with reports from South Africa to southern Europe, and from western Africa to southern China. This study presents a global compendium of *Culicoides imicola* occurrence between 1943 and 2018, reflecting the most recently compiled and harmonized global dataset derived from peer-reviewed literature. The procedures used in producing the data, as well as the geo-coding methods, database management and technical validation procedures are described. The study provides an updated and comprehensive global database of *C*. *imicola* occurrence, consisting of 1 039 geo-coded records from 50 countries. The datasets can be used for risk mapping of the diseases transmitted by *C*. *imicola* as well as to develop the global habitat suitability for the vector.

## Abstract (summary)

**Table:** 

Measurement(s)	publication • Disease Vector
Technology Type(s)	digital curation
Factor Type(s)	year • geographic location
Sample Characteristic - Organism	Culicoides imicola
Sample Characteristic - Location	Earth (planet)

Machine-accessible metadata file describing the reported data: 10.6084/m9.figshare.9804509

## Background & Summary

*Culicoides imicola* Kieffer (Diptera: Ceratopogonidae) is a globally widespread species that vectors the agents of many important viral diseases of veterinary importance such as Bluetongue^1–3^, African Horse Sickness (AHS)^4,5^, and Schmallenberg^6^. Bluetongue (BT) is a viral disease that affects ruminants and the etiological agent has at least 27 different serotypes^7–9^. Historically, BT was enzootic in tropical regions of the world, but in recent years it has expanded its distribution markedly. The disease has become a concern in areas that experience a temperate climate, particularly in Europe. This expanding disease distribution is mainly facilitated by northward distribution of the infected *Culicoides* species mainly *C*. *imicola* and availability of competent and efficient vectors such as *C*. *obsoletus* and *C*. *pulicaris*^7,10^. The 1998 incursion and emergence of bluetongue virus in Southern and Eastern Europe were manly associated with *C*. *imicola*, while the 2006 incursion of Northern and Western Europe^7^ was mainly associated with *C*. *obsoletus* and *C*. *pulicaris*.

AHS is native to sub-Saharan Africa^4^. It is an infectious disease considered to be the most lethal viral disease of equines, especially in horses^4,11^. The recent emergence of the two Culicoides-borne diseases (BT and Schmallenberg) in Europe has raised a concern for the potential introduction and further spread of AHS virus in temperate parts of the world as well^11^.

Although *C*. *obsoletus* and *C*. *pulicaris* are considered as main vectors for Schmallenberg, experimental infection on field collected *C*. *imicola* provided evidence of high efficiency for Schmallenberg virus infection and transmission by *C*. *imicola* as well^6^. Schmallenberg virus is a very recently emerged virus first identified in North Rhine-Westphalia, Germany, during the summer of 2011^12^ and since then it has spread across Europe causing congenital deformities in the offspring of infected adult ruminants^13^.

The recent emergence of Culicoides-borne diseases highlights large knowledge gaps on the biology and ecology of the vectors. Since the emergence of BT and Schmallenberg virus, Culicoides surveillance efforts have doubled. Thus, it is important and timely to expand the effort of Guichard *et al*.^14^ and update the global Culicoides occurrence record. With these research gaps in mind, this study compiled the global occurrences of *C*. *imicola* based on the dataset provided by Guichard *et al*.^14^ and literature published since 1^st^ January 2014 and created the largest currently available standardized up-to-date georeferenced global dataset for the vector, containing 1 039 occurrence records.

## Methods

### Literature search and data extraction

**Fig. 1:**
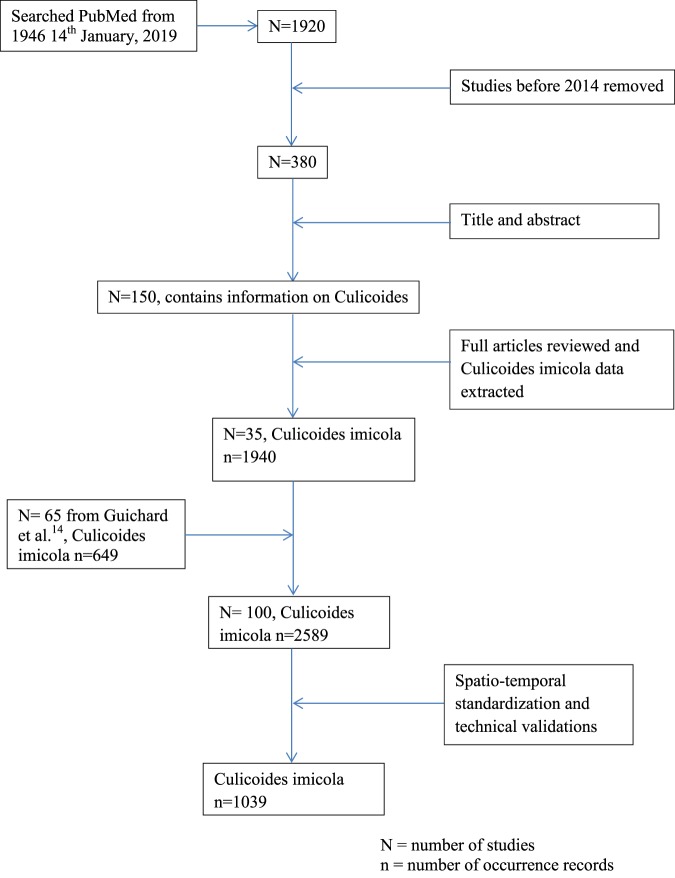
Flow chart of literature search and data extraction.

PubMed (http://www.ncbi.nlm.nih.gov) was searched using the term ‘*Culicoides imicola*’ OR ‘Ceratopogonidae’. Automatic inclusion of all pseudonyms in the searches was guaranteed by using the Medical Subject Headings (MeSH) term technology of the PubMed citation archive (http://www.nlm.nih.gov/mesh). The literature search was last updated on 14^th^ January 2019, which resulted in a collection of 1 920 articles. However, a geo‐database of 649 occurrences of *C*. *imicola* compiled by Guichard *et al*.^14^ from 65 articles^5,15–78^ covering 1943 to 2010 (1959 to 2014 by publication year) was obtained from the authors. Thus, in this study a literature search for the period 1^st^ January 2014 till 14^th^ January 2019^4,6,79–111^ was combined with existing data points obtained from Guichard *et al*.^14^ for the period 1959 to 2014. The search retrieved a total of 380 articles published since 1^st^ January 2014 and the titles and abstracts of those articles were screened and those not fitting the criteria: 1) no mention of the vector species; and 2) data from experimental study were removed. After literature searching and initial selections, 150 eligible full-text articles were downloaded and examined in detail to filter those meeting the following criteria: 1) the coordinates of field sites were reported or could be retrieved from Google earth using the reported location information, and 2) occurrence of *C*. *imicola* was reported. Therefore, each entry was checked for site coordinates, occurrence of the target species, and other information if available. Subsequently, the geo-location of the vector was extracted from a total of 35 articles meeting all the criteria (Fig. 1). Each article was thoroughly reviewed and all important information was extracted: site location, site name, year of data collection and other information, and confirmed *C*. *imicola* occurrences within these articles were entered into the database. Occurrences were classified as confirmed when the article clearly stated the presence of the vector at a specific time in a specific location.

### Geo-coding of data

The occurrence coordinates (longitude and latitude) of *C*. *imicola* was extracted from each articles and whenever the coordinates were not provided in the articles or supporting information of the articles, the study site name together with all contextual information as well as alternative spelling of site name was used to determine its coordinates using Google Earth (http://www.google.co.uk/intl/en_uk/earth). When two locations have the same name and different geolocations, both the location name and occurrence coordinates were provided. All data points were then linked to the FAO Global Administrative Unit Layer (GAUL) system (http://www.fao.org/geonetwork)^112^ by using a join attributes by location tool in QGIS Version 3.4 (https://qgis.org/).

## Data Records

R (https://cran.r-project.org/), QGIS (https://qgis.org/), Mendeley Desktop http://www.mendeley.com/, and Microsoft Excel were the software packages used to manage, store and analyze the database. The dataset is saved in a comma-delimited (.csv), format and can be imported into a variety of Statistical and GIS software programs. The data records described in this paper are publicly and freely available on Figshare^113^. There are 2 589 entries (before technical validations) and 1 039 entries (after technical validations) with information in 8 columns (Table 1) in the dataset. The spatial thinning procedure was provided under technical validation section. In the data, the rows represent a single occurrence record (one or more *C*. *imicola* occurrences in the same unique location within a single calendar year). The fields contained in the database are described in Table 1.

**Table 1:** Description of attributes and columns in the dataset.

Attribute	Column #	Column name	Unit	Note
Reference	1	Reference	—	Author names and publication year of the article from which the occurrence record is extracted
Site location	2	UNREGION2	—	The name of UN region 2 within which the occurrence lies (Global Administrative Unit Layers (GAUL) system)
	3	UNREGION1	—	The name of the UN region 1 within which the occurrence lies (Global Administrative Unit Layers (GAUL) system)
	4	Country		Name of the country within which the occurrence lies (Global Administrative Unit Layers (GAUL) system).
	6	Site name		Name of the site where the occurrence lies
	7	Longitude	Degree East/West	The longitudinal coordinate of the occurrence point (WGS1984 Datum)
	8	Latitude	Degree North/South	The latitudinal coordinate of the occurrence point (WGS1984 Datum)
Year		Year		Year of *C*. *imicola* occurrence

## Technical Validation

To ensure the accuracy and validity of the occurrence records, a technical validation was performed. Firstly, a 5 km × 5 km resolution landcover raster was used to ensure all occurrences were positioned on a valid land pixel. Based on the reported coordinates some sites (n = 96) fell on water bodies. This was probably due to the precision of the longitude and latitude values since these sites were all in peri-coastal locations. Thus, from 2 589 occurrence points 96 were removed from the database.

Further, as the database was compiled from different sources and over many years, it was important to standardize the data entries such that identical locations which may have been geo-positioned slightly differently were given the same unique identifier. The present dataset is heavily clustered in Europe and Southern Africa, with a high degree of aggregation in Spain, Portugal, Italy and South Africa compared to elsewhere. Consequently, it was important to spatially thin the occurrence records. The spatial thinning was performed using R package spThin^114^ with the use of the following parameters: “*thin*.*par*” (the distance between occurrence records in kilometers) and “*reps*” (the number of times to repeat the thinning process). In the thinning process, the distance (in kilometers) between occurrence records was set to 5 km (meaning if occurrence records lay within the same 5 km × 5 km pixel within a global grid only one record was retained) and the number of times to repeat the thinning process was set at 100. As a result, the 2 493 occurrence points were reduced down to 1 039.

**Fig. 2:**
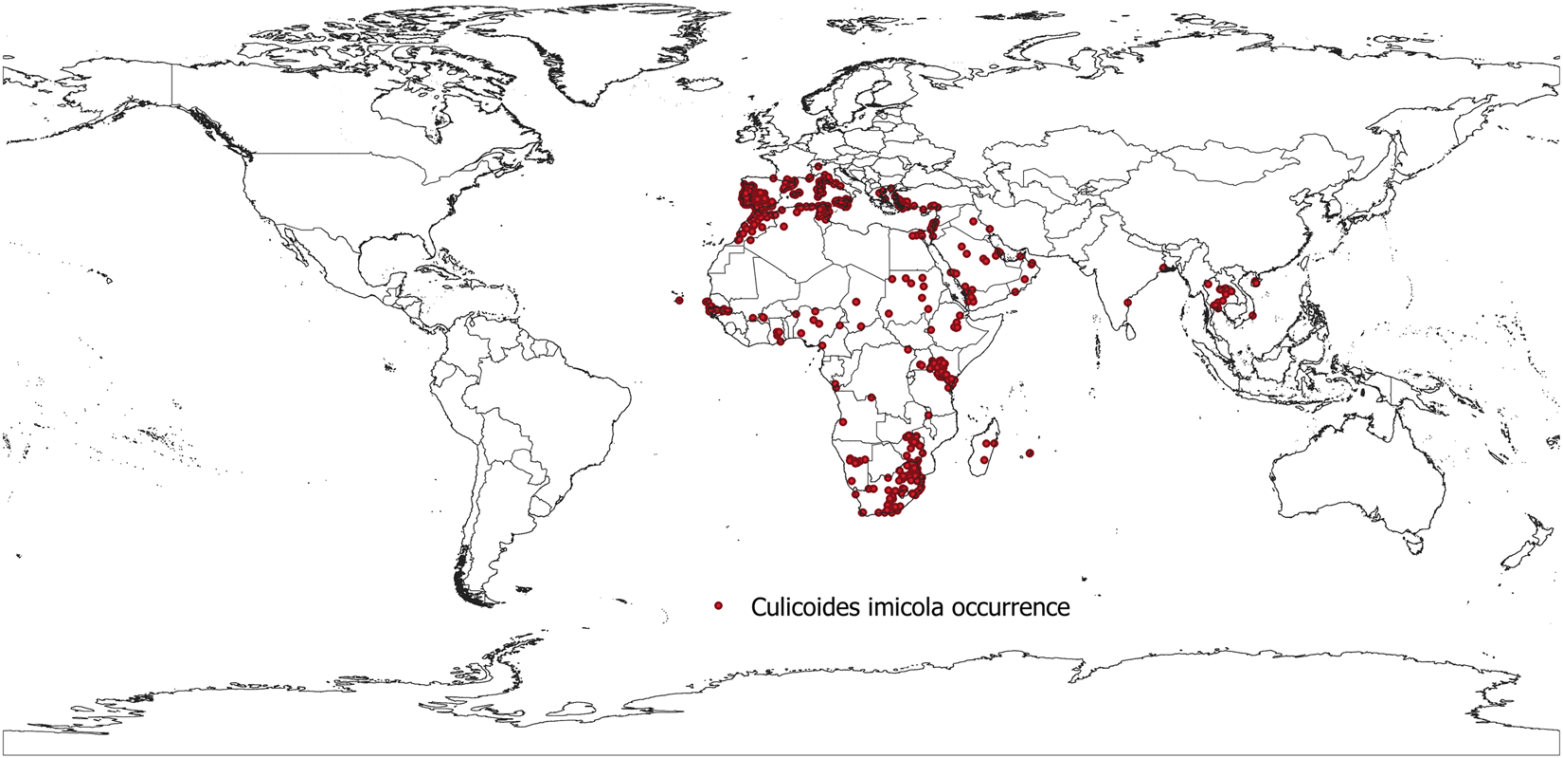
Map of occurrence points for Culicoides imicola.

The resulting database consists of 2 589 (before technical validations) and 1 039 (after technical validations) geo-positioned occurrences of *C*. *imicola* spanning 50 countries worldwide, disaggregated by continent, region, and country (Table 2). The data before technical validations includes the 96 occurrence points that fell on water bodies as well. In Fig. 2 the global geographical distribution of *C*. *imicola* is displayed.

**Table 2:** *Culicoides imicola* occurrence records by UN region.

Continent (UN region 2)	Region (UN region 1)	Number of *C*. *imicola* before technical validation	Number of *C*. *imicola* after technical validation
Africa	Eastern Africa	104	84
	Middle Africa	10	10
	Northern Africa	86	81
	Southern Africa	153	96
	Western Africa	71	40
Americas	Caribbean	0	0
	Central America	0	0
	Northern America	0	0
	South America	0	0
Asia	Central Asia	0	0
	Eastern Asia	2	2
	South-Eastern Asia	20	18
	Southern Asia	5	5
	Western Asia	100	63
Europe	Eastern Europe	0	0
	Northern Europe	0	0
	Southern Europe	1998	615
	Western Europe	40	25
Oceania	Melanesia	0	0
	Micronesia	0	0
	Australia and New Zealand	0	0
	Polynesia	0	0
Total		2589	1039

## Usage Notes

The database described here can be used to investigate the spatial and temporal distribution of *C*. *imicola*. The data are most appropriate for applications at global and continental scales. It is known that *C*. *imicola* and the diseases transmitted by the vector were previously known to be a problem of Africa. However, due to the recent spread of the species to Europe and other parts of the world^115–117^, this data could support improved modelling of new locations at high-risk of experiencing the occurrence of the vector as well as the diseases transmitted by it. The database after technical validations could be used to develop suitability and risk maps at global, continental, and regional scales. On the other hand, for local scale suitability and risk mapping, the database before technical validations could be used.

There are differences in the number of published studies and the availability of occurrence data by continent and region. Continental and regional biases in the density of occurrence records are apparent, and likely reflect differences in the level of surveillance. Due to the recent occurrence of Bluetongue and Schmallenberg viruses in Europe, substantial numbers of surveys have been conducted in Europe, and thus large numbers of recent occurrence records were from Europe. Many occurrence records were also obtained from Southern Africa. From 1 550 points thinned from the database during validations, 1 440 (92.9%) is from Southern Europe and Southern Africa. Thus, researchers using the technically unvalidated database would need to take into account geographical sampling bias.

## Data Availability

There is no custom code produced during the collection and validation of this dataset.
